# Ethyl 2,5-di-*tert*-butyl-5-eth­oxy-4-oxo-4,5-di­hydro-1*H*-pyrrole-3-carboxyl­ate

**DOI:** 10.1107/S1600536813012282

**Published:** 2013-05-11

**Authors:** Gerald M. Rosen, Sukumaran Muralidharan, Peter Y. Zavalij, Steven Fletcher, Joseph P. Y. Kao

**Affiliations:** a Department of Pharmaceutical Sciences, School of Pharmacy, University of Maryland, Baltimore, MD 21201, USA; b Center for Biomedical Engineering and Technology, School of Medicine, University of Maryland, Baltimore, MD 21201, USA; c Department of Chemistry and Biochemistry, University of Maryland, College Park, MD 20742, USA; d Department of Physiology, School of Medicine, University of Maryland, Baltimore, MD 21201, USA

## Abstract

The title compound, C_17_H_29_NO_4_, contains a chiral center and crystallizes as a racemate. The asymmetric unit consists of two non-equivalent mol­ecules, in which the carbeth­oxy groups have markedly different orientations [C(=O)CC(OEt)=O torsion angles = 59.3 (2) and 156.0 (2)°]. In the crystal, mol­ecules form chains along [101] through N—H⋯O hydrogen bonds.

## Related literature

The title compound resulted from an attempt to devise a more efficient synthesis of diethyl 2,5,-di(*tert*-but­yl)-1-hy­droxy­pyrrole-3,4-di­carboxyl­ate, a precursor to an aromatic nitroxide (Ramasseul & Rassat, 1970 ▶). For related synthetic procedures, see also: Riplinger *et al.* (2009 ▶); Travis *et al.* (2003 ▶).
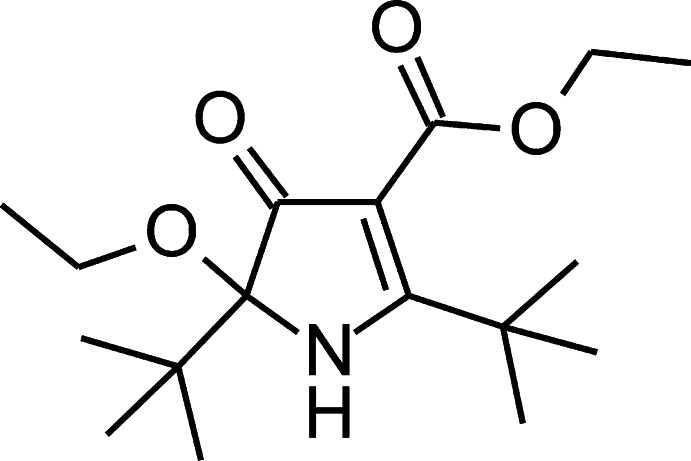


## Experimental

### 

#### Crystal data

C_17_H_29_NO_4_
*M* *_r_* = 311.41
Monoclinic, 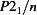

*a* = 9.9187 (7) Å
*b* = 16.3916 (11) Å
*c* = 22.2115 (15) Å
β = 92.5822 (11)°
*V* = 3607.6 (4) Å^3^
*Z* = 8
Mo *K*α radiation
μ = 0.08 mm^−1^
*T* = 150 K
0.42 × 0.40 × 0.33 mm

#### Data collection

Bruker SMART APEXII diffractometer
Absorption correction: multi-scan (*SADABS*; Sheldrick, 1996 ▶) *T*_min_ = 0.887, *T*_max_ = 0.974
40948 measured reflections
6356 independent reflections
5812 reflections with *I* > 2σ(*I*)
*R*_int_ = 0.018

#### Refinement

*R*[*F* ^2^ > 2σ(*F* ^2^)] = 0.039
*wR*(*F* ^2^) = 0.077
*S* = 1.00
6356 reflections
441 parameters
H-atom parameters constrained
Δρ_max_ = 0.31 e Å^−3^
Δρ_min_ = −0.25 e Å^−3^

### 

Data collection: *APEX2* (Bruker, 2010 ▶); cell refinement: *APEX2* and *SAINT* (Bruker, 2010 ▶); data reduction: *SAINT*; program(s) used to solve structure: *SHELXS97* (Sheldrick, 2008 ▶); program(s) used to refine structure: *SHELXL2012* (Sheldrick, 2008 ▶); molecular graphics: *XSHELL* (Bruker, 2010 ▶) and *Mercury* (Macrae *et al.*, 2008 ▶); software used to prepare material for publication: *publCIF* (Westrip, 2010 ▶).

## Supplementary Material

Crystal structure: contains datablock(s) I, global. DOI: 10.1107/S1600536813012282/ld2098sup1.cif

Structure factors: contains datablock(s) I. DOI: 10.1107/S1600536813012282/ld2098Isup2.hkl

Supplementary material file. DOI: 10.1107/S1600536813012282/ld2098Isup3.cml

Additional supplementary materials:  crystallographic information; 3D view; checkCIF report

## Figures and Tables

**Table 1:** Hydrogen-bond geometry (Å, °)

*D*—H⋯*A*	*D*—H	H⋯*A*	*D*⋯*A*	*D*—H⋯*A*
N4*A*—H4*A*⋯O1*B* ^i^	0.832 (16)	2.175 (16)	3.0009 (15)	171.8 (14)
N4*B*—H4*B*⋯O1*A*	0.862 (16)	2.156 (17)	3.0089 (15)	170.4 (15)

Symmetry code: (i) 
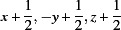
.

**Fig. 1.**
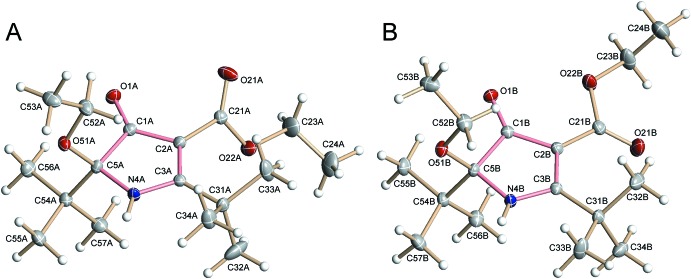
Molecular structure of the title compound showing nonequivalent molecules A and B, with non-hydrogen atoms labeled. Displacement ellipsoids are shown at the 30% probability level. Note that molecule B was inverted in the center of symmetry to facilitate comparison with molecule A.

**Fig. 2.**
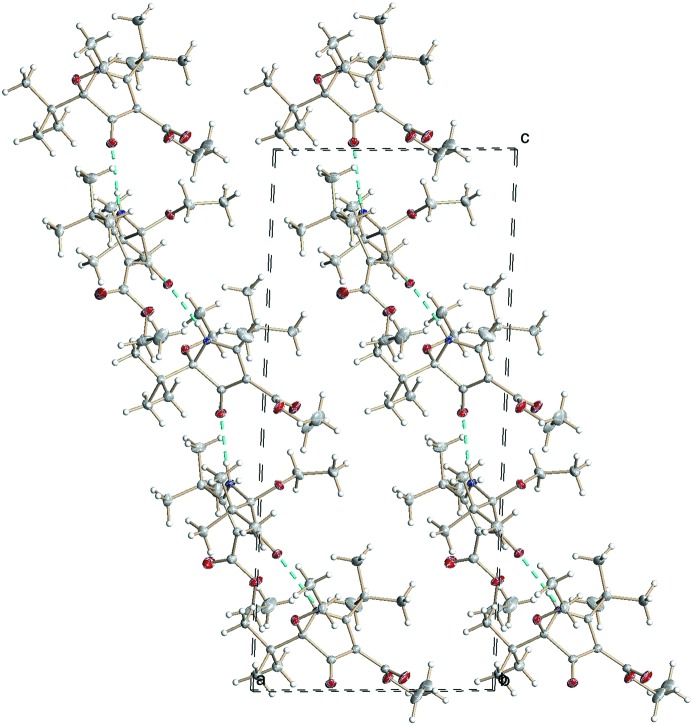
Packing of molecules A and B shown along b axis depicting chains of molecules linked through N—H···O hydrogen bonds (light blue dashed lines) in the 101 direction. Only half of the unit cell is shown along b for visual clarity.

## supplementary crystallographic information

### Comment

The title compound resulted from an attempt to devise a more efficient synthesis of diethyl 2,5,-di(*tert*-butyl)-1-hydroxypyrrole-3,4-dicarboxylate, a precursor to an aromatic nitroxide (Ramasseul & Rassat, 1970). The molecular structure is shown in Fig. 1. Despite the steric bulk of the substituents, the pyrroline rings are planar (the r.m.s. deviations of the atoms in the rings containing N4A and N4B are 0.020 and 0.036 Å, respectively). In the crystal, N—H···O hydrogen bonds link the molecules into chains (Fig. 2).

### Experimental

The title comnpund was prepared in three steps from the known compound, ethyl 2,5-di(*tert*-butyl)-1-hydroxypyrrole-3-carboxylate (Ramasseul & Rassat, 1970; Riplinger *et al.*, 2009): 1) *O*-benzylation, 2) Vilsmeier formylation of the 4 position, and 3) oxidation by Oxone in ethanol (Travis *et al.* 2003). Instead of the expected conversion of the 4-formyl group into the corresponding ethyl ester, Oxone treatment resulted in the title compound. After purification of the product by flash chromatography, single crystals were obtained by recrystallization from ethyl acetate.

### Refinement

Position of all H atoms was calculated from geometric considerations. H atoms were refined as riding on the attached C atoms, except H atoms in N—H groups which were freely refined. Orientation of CH_3_ groups was optimized. For all H atoms *U*_iso_ were refined but constrained to be equal within CH_3_ groups.

### Crystal data

**Table:**

C_17_H_29_NO_4_	*F*(000) = 1360
*M**_r_* = 311.41	*D*_x_ = 1.147 Mg m^−^^3^
Monoclinic, *P*2_1_/*n*	Mo *K*α radiation, λ = 0.71073 Å
*a* = 9.9187 (7) Å	Cell parameters from 26267 reflections
*b* = 16.3916 (11) Å	θ = 2.2–32.0°
*c* = 22.2115 (15) Å	µ = 0.08 mm^−^^1^
β = 92.5822 (11)°	*T* = 150 K
*V* = 3607.6 (4) Å^3^	Prism, colourless
*Z* = 8	0.42 × 0.40 × 0.33 mm

### Data collection

**Table:**

Bruker SMART APEXII diffractometer	6356 independent reflections
Radiation source: sealed tube	5812 reflections with *I* > 2σ(*I*)
Graphite monochromator	*R*_int_ = 0.018
Detector resolution: 8.333 pixels mm^-1^	θ_max_ = 25.0°, θ_min_ = 2.2°
φ and ω scans	*h* = −11→11
Absorption correction: multi-scan (*SADABS*; Sheldrick, 1996)	*k* = −19→19
*T*_min_ = 0.887, *T*_max_ = 0.974	*l* = −26→26
40948 measured reflections	

### Refinement

**Table:**

Refinement on *F*^2^	Primary atom site location: structure-invariant direct methods
Least-squares matrix: full	Secondary atom site location: difference Fourier map
*R*[*F*^2^ > 2σ(*F*^2^)] = 0.039	Hydrogen site location: inferred from neighbouring sites
*wR*(*F*^2^) = 0.077	H-atom parameters constrained
*S* = 1.00	*w* = 1/[σ^2^(*F*_o_^2^) + (0.010*P*)^2^ + 2.698*P*] where *P* = (*F*_o_^2^ + 2*F*_c_^2^)/3
6356 reflections	(Δ/σ)_max_ < 0.001
441 parameters	Δρ_max_ = 0.31 e Å^−^^3^
0 restraints	Δρ_min_ = −0.25 e Å^−^^3^

### Special details

**Table:**

Geometry. All e.s.d.'s (except the e.s.d. in the dihedral angle between two l.s. planes) are estimated using the full covariance matrix. The cell e.s.d.'s are taken into account individually in the estimation of e.s.d.'s in distances, angles and torsion angles; correlations between e.s.d.'s in cell parameters are only used when they are defined by crystal symmetry. An approximate (isotropic) treatment of cell e.s.d.'s is used for estimating e.s.d.'s involving l.s. planes.
Refinement. Refinement of *F*^2^ against ALL reflections. The weighted *R*-factor *wR* and goodness of fit *S* are based on *F*^2^, conventional *R*-factors *R* are based on *F*, with *F* set to zero for negative *F*^2^. The threshold expression of *F*^2^ > σ(*F*^2^) is used only for calculating *R*-factors(gt) *etc*. and is not relevant to the choice of reflections for refinement. *R*-factors based on *F*^2^ are statistically about twice as large as those based on *F*, and *R*– factors based on ALL data will be even larger.

### Fractional atomic coordinates and isotropic or equivalent isotropic displacement parameters (Å^2^)

**Table:**

	*x*	*y*	*z*	*U*_iso_*/*U*_eq_	
O1A	0.17249 (10)	0.17307 (6)	0.51228 (4)	0.0309 (2)	
C1A	0.17385 (13)	0.21856 (8)	0.55631 (6)	0.0226 (3)	
C2A	0.07337 (13)	0.27417 (8)	0.57559 (6)	0.0222 (3)	
C3A	0.11821 (13)	0.30765 (8)	0.63052 (6)	0.0212 (3)	
N4A	0.24234 (11)	0.28082 (7)	0.64688 (5)	0.0235 (2)	
H4A	0.2852 (15)	0.2948 (9)	0.6782 (7)	0.026 (4)*	
C5A	0.29647 (13)	0.22476 (8)	0.60283 (6)	0.0221 (3)	
C21A	−0.05218 (14)	0.28839 (9)	0.53915 (6)	0.0243 (3)	
O21A	−0.13140 (11)	0.23672 (7)	0.52273 (5)	0.0443 (3)	
O22A	−0.06552 (11)	0.36716 (6)	0.52314 (5)	0.0369 (3)	
C23A	−0.18864 (19)	0.38801 (12)	0.48838 (9)	0.0543 (5)	
H23A	−0.2621	0.3501	0.4983	0.077 (5)*	
H23B	−0.1736	0.3835	0.4447	0.077 (5)*	
C24A	−0.2263 (2)	0.47213 (13)	0.50333 (10)	0.0641 (6)	
H24A	−0.2423	0.4759	0.5465	0.094 (5)*	
H24B	−0.3086	0.4874	0.4800	0.094 (5)*	
H24C	−0.1529	0.5092	0.4935	0.094 (5)*	
C31A	0.04558 (14)	0.36525 (9)	0.67184 (6)	0.0271 (3)	
C32A	0.0885 (2)	0.45196 (11)	0.65724 (10)	0.0612 (6)	
H32A	0.1860	0.4577	0.6653	0.066 (4)*	
H32B	0.0406	0.4905	0.6824	0.066 (4)*	
H32C	0.0664	0.4635	0.6146	0.066 (4)*	
C33A	−0.10814 (16)	0.35611 (13)	0.66379 (7)	0.0467 (5)	
H33A	−0.1383	0.3763	0.6239	0.070 (4)*	
H33B	−0.1517	0.3877	0.6950	0.070 (4)*	
H33C	−0.1326	0.2985	0.6674	0.070 (4)*	
C34A	0.08294 (17)	0.34531 (12)	0.73789 (7)	0.0416 (4)	
H34A	0.0611	0.2882	0.7460	0.054 (3)*	
H34B	0.0318	0.3808	0.7641	0.054 (3)*	
H34C	0.1798	0.3543	0.7458	0.054 (3)*	
O51A	0.33071 (9)	0.14932 (6)	0.62981 (4)	0.0267 (2)	
C52A	0.21923 (15)	0.10469 (10)	0.65294 (7)	0.0350 (4)	
H52A	0.1677	0.0771	0.6196	0.050 (4)*	
H52B	0.1577	0.1424	0.6731	0.050 (4)*	
C53A	0.27518 (17)	0.04306 (10)	0.69693 (8)	0.0410 (4)	
H53A	0.3338	0.0051	0.6762	0.059 (3)*	
H53B	0.2009	0.0128	0.7141	0.059 (3)*	
H53C	0.3276	0.0709	0.7293	0.059 (3)*	
C54A	0.42581 (14)	0.25865 (9)	0.57484 (6)	0.0251 (3)	
C55A	0.53624 (14)	0.27112 (10)	0.62480 (7)	0.0326 (3)	
H55A	0.6202	0.2880	0.6068	0.041 (3)*	
H55B	0.5513	0.2199	0.6468	0.041 (3)*	
H55C	0.5077	0.3134	0.6527	0.041 (3)*	
C56A	0.47769 (15)	0.19853 (10)	0.52844 (7)	0.0344 (3)	
H56A	0.4133	0.1957	0.4937	0.045 (3)*	
H56B	0.4875	0.1444	0.5468	0.045 (3)*	
H56C	0.5654	0.2171	0.5151	0.045 (3)*	
C57A	0.39392 (16)	0.34066 (9)	0.54419 (7)	0.0337 (3)	
H57A	0.4765	0.3632	0.5282	0.043 (3)*	
H57B	0.3585	0.3786	0.5737	0.043 (3)*	
H57C	0.3264	0.3325	0.5112	0.043 (3)*	
O1B	−0.08469 (10)	0.15836 (6)	0.25177 (4)	0.0286 (2)	
C1B	−0.00038 (13)	0.13501 (8)	0.29014 (6)	0.0219 (3)	
C2B	0.08310 (13)	0.06294 (8)	0.29281 (6)	0.0222 (3)	
C3B	0.14805 (13)	0.06053 (8)	0.35069 (6)	0.0215 (3)	
N4B	0.12100 (11)	0.12751 (7)	0.38204 (5)	0.0226 (2)	
H4B	0.1407 (15)	0.1351 (10)	0.4198 (7)	0.030 (4)*	
C5B	0.03325 (13)	0.18511 (8)	0.34818 (6)	0.0215 (3)	
C21B	0.09972 (14)	0.00705 (8)	0.24244 (6)	0.0269 (3)	
O21B	0.19358 (12)	−0.03802 (8)	0.23510 (5)	0.0482 (3)	
O22B	−0.00323 (12)	0.01369 (7)	0.20099 (5)	0.0411 (3)	
C23B	0.0058 (2)	−0.03658 (11)	0.14761 (8)	0.0544 (5)	
H23C	0.1019	−0.0454	0.1392	0.069 (5)*	
H23D	−0.0375	−0.0079	0.1126	0.069 (5)*	
C24B	−0.0613 (2)	−0.11702 (13)	0.15562 (11)	0.0688 (7)	
H24D	−0.0151	−0.1468	0.1888	0.083 (4)*	
H24E	−0.0571	−0.1488	0.1184	0.083 (4)*	
H24F	−0.1559	−0.1083	0.1650	0.083 (4)*	
C31B	0.23707 (14)	−0.00578 (8)	0.37980 (6)	0.0260 (3)	
C32B	0.37584 (15)	−0.00186 (10)	0.35174 (8)	0.0359 (4)	
H32D	0.3650	−0.0109	0.3082	0.050 (3)*	
H32E	0.4348	−0.0441	0.3698	0.050 (3)*	
H32F	0.4162	0.0519	0.3594	0.050 (3)*	
C33B	0.25616 (19)	0.00727 (11)	0.44792 (7)	0.0462 (5)	
H33D	0.3026	0.0592	0.4558	0.056 (3)*	
H33E	0.3103	−0.0374	0.4656	0.056 (3)*	
H33F	0.1678	0.0083	0.4660	0.056 (3)*	
C34B	0.17217 (16)	−0.08995 (9)	0.36926 (8)	0.0393 (4)	
H34D	0.0832	−0.0910	0.3867	0.056 (3)*	
H34E	0.2297	−0.1320	0.3884	0.056 (3)*	
H34F	0.1621	−0.1006	0.3259	0.056 (3)*	
O51B	−0.08119 (10)	0.20552 (6)	0.37952 (4)	0.0272 (2)	
C52B	−0.15794 (16)	0.13701 (10)	0.39956 (7)	0.0363 (4)	
H52C	−0.1222	0.1185	0.4396	0.045 (3)*	
H52D	−0.1516	0.0911	0.3708	0.045 (3)*	
C53B	−0.30206 (16)	0.16360 (11)	0.40323 (7)	0.0422 (4)	
H53D	−0.3068	0.2104	0.4305	0.056 (3)*	
H53E	−0.3557	0.1186	0.4186	0.056 (3)*	
H53F	−0.3379	0.1792	0.3630	0.056 (3)*	
C54B	0.10890 (14)	0.26597 (8)	0.33434 (6)	0.0249 (3)	
C55B	0.16242 (17)	0.30456 (9)	0.39357 (7)	0.0373 (4)	
H55D	0.2311	0.2689	0.4127	0.047 (3)*	
H55E	0.0879	0.3117	0.4206	0.047 (3)*	
H55F	0.2025	0.3578	0.3852	0.047 (3)*	
C56B	0.22690 (15)	0.24875 (9)	0.29401 (7)	0.0332 (3)	
H56D	0.2756	0.2996	0.2868	0.044 (3)*	
H56E	0.1923	0.2263	0.2555	0.044 (3)*	
H56F	0.2884	0.2094	0.3139	0.044 (3)*	
C57B	0.01182 (16)	0.32582 (9)	0.30168 (7)	0.0338 (3)	
H57D	0.0608	0.3755	0.2916	0.043 (3)*	
H57E	−0.0616	0.3396	0.3280	0.043 (3)*	
H57F	−0.0258	0.3005	0.2646	0.043 (3)*	

### Atomic displacement parameters (Å^2^)

**Table:**

	*U*^11^	*U*^22^	*U*^33^	*U*^12^	*U*^13^	*U*^23^
O1A	0.0366 (6)	0.0310 (6)	0.0247 (5)	0.0040 (4)	−0.0033 (4)	−0.0092 (4)
C1A	0.0264 (7)	0.0216 (7)	0.0196 (7)	0.0003 (5)	−0.0007 (5)	0.0000 (5)
C2A	0.0231 (7)	0.0216 (7)	0.0216 (7)	0.0008 (5)	−0.0017 (5)	−0.0005 (5)
C3A	0.0214 (7)	0.0204 (7)	0.0218 (7)	−0.0006 (5)	0.0001 (5)	0.0003 (5)
N4A	0.0216 (6)	0.0298 (6)	0.0187 (6)	0.0029 (5)	−0.0041 (5)	−0.0065 (5)
C5A	0.0240 (7)	0.0217 (7)	0.0205 (6)	0.0046 (5)	−0.0007 (5)	−0.0014 (5)
C21A	0.0244 (7)	0.0286 (7)	0.0196 (6)	0.0014 (6)	−0.0001 (5)	−0.0009 (6)
O21A	0.0400 (6)	0.0415 (7)	0.0494 (7)	−0.0116 (5)	−0.0210 (5)	0.0072 (5)
O22A	0.0368 (6)	0.0275 (6)	0.0449 (6)	0.0040 (5)	−0.0167 (5)	0.0044 (5)
C23A	0.0488 (11)	0.0477 (11)	0.0635 (12)	0.0136 (9)	−0.0300 (9)	0.0058 (9)
C24A	0.0592 (13)	0.0605 (13)	0.0713 (14)	0.0276 (11)	−0.0126 (11)	0.0149 (11)
C31A	0.0270 (7)	0.0279 (8)	0.0265 (7)	0.0043 (6)	0.0015 (6)	−0.0065 (6)
C32A	0.0923 (16)	0.0274 (9)	0.0665 (13)	−0.0017 (10)	0.0317 (12)	−0.0142 (9)
C33A	0.0293 (8)	0.0763 (13)	0.0346 (9)	0.0138 (8)	0.0013 (7)	−0.0165 (9)
C34A	0.0383 (9)	0.0604 (11)	0.0259 (8)	0.0128 (8)	−0.0003 (7)	−0.0133 (8)
O51A	0.0229 (5)	0.0251 (5)	0.0322 (5)	0.0034 (4)	0.0005 (4)	0.0062 (4)
C52A	0.0272 (8)	0.0334 (8)	0.0445 (9)	−0.0021 (6)	0.0012 (7)	0.0089 (7)
C53A	0.0390 (9)	0.0373 (9)	0.0460 (10)	−0.0103 (7)	−0.0058 (7)	0.0130 (8)
C54A	0.0244 (7)	0.0270 (7)	0.0241 (7)	0.0022 (6)	0.0010 (5)	0.0000 (6)
C55A	0.0230 (7)	0.0432 (9)	0.0317 (8)	−0.0012 (6)	0.0008 (6)	−0.0008 (7)
C56A	0.0325 (8)	0.0402 (9)	0.0312 (8)	0.0031 (7)	0.0086 (6)	−0.0041 (7)
C57A	0.0346 (8)	0.0302 (8)	0.0360 (8)	−0.0030 (7)	0.0006 (7)	0.0056 (7)
O1B	0.0281 (5)	0.0305 (5)	0.0265 (5)	0.0038 (4)	−0.0059 (4)	0.0023 (4)
C1B	0.0217 (7)	0.0224 (7)	0.0215 (7)	−0.0017 (5)	0.0015 (5)	0.0022 (5)
C2B	0.0229 (7)	0.0210 (7)	0.0226 (7)	−0.0001 (5)	0.0005 (5)	−0.0004 (5)
C3B	0.0212 (6)	0.0193 (7)	0.0242 (7)	−0.0010 (5)	0.0027 (5)	0.0008 (5)
N4B	0.0286 (6)	0.0206 (6)	0.0182 (6)	0.0036 (5)	−0.0028 (5)	−0.0004 (5)
C5B	0.0231 (7)	0.0200 (7)	0.0215 (7)	0.0045 (5)	0.0011 (5)	0.0013 (5)
C21B	0.0316 (8)	0.0226 (7)	0.0263 (7)	−0.0011 (6)	−0.0006 (6)	−0.0011 (6)
O21B	0.0462 (7)	0.0523 (8)	0.0452 (7)	0.0198 (6)	−0.0059 (5)	−0.0231 (6)
O22B	0.0543 (7)	0.0367 (6)	0.0309 (6)	0.0120 (5)	−0.0151 (5)	−0.0131 (5)
C23B	0.0871 (15)	0.0425 (10)	0.0315 (9)	0.0176 (10)	−0.0197 (9)	−0.0156 (8)
C24B	0.0708 (14)	0.0510 (12)	0.0817 (16)	0.0053 (11)	−0.0278 (12)	−0.0296 (11)
C31B	0.0281 (7)	0.0209 (7)	0.0286 (7)	0.0049 (6)	−0.0012 (6)	0.0022 (6)
C32B	0.0263 (8)	0.0337 (8)	0.0474 (9)	0.0042 (6)	−0.0020 (7)	0.0064 (7)
C33B	0.0643 (12)	0.0417 (10)	0.0316 (9)	0.0266 (9)	−0.0074 (8)	0.0044 (7)
C34B	0.0358 (9)	0.0237 (8)	0.0582 (11)	0.0023 (7)	−0.0023 (8)	0.0095 (7)
O51B	0.0286 (5)	0.0240 (5)	0.0299 (5)	0.0040 (4)	0.0092 (4)	0.0015 (4)
C52B	0.0386 (9)	0.0318 (8)	0.0398 (9)	−0.0050 (7)	0.0150 (7)	−0.0002 (7)
C53B	0.0333 (9)	0.0588 (11)	0.0349 (9)	−0.0081 (8)	0.0063 (7)	−0.0108 (8)
C54B	0.0296 (7)	0.0191 (7)	0.0262 (7)	0.0005 (6)	0.0017 (6)	0.0005 (6)
C55B	0.0505 (10)	0.0262 (8)	0.0348 (8)	−0.0063 (7)	−0.0029 (7)	−0.0040 (7)
C56B	0.0326 (8)	0.0281 (8)	0.0394 (9)	−0.0036 (6)	0.0083 (7)	0.0023 (7)
C57B	0.0418 (9)	0.0218 (7)	0.0379 (8)	0.0049 (6)	0.0017 (7)	0.0056 (6)

### Geometric parameters (Å, º)

**Table:**

O1A—C1A	1.2294 (16)	O1B—C1B	1.2279 (16)
C1A—C2A	1.4304 (19)	C1B—C2B	1.4424 (18)
C1A—C5A	1.5634 (18)	C1B—C5B	1.5520 (18)
C2A—C3A	1.3925 (18)	C2B—C3B	1.4123 (18)
C2A—C21A	1.4728 (18)	C2B—C21B	1.4612 (19)
C3A—N4A	1.3420 (17)	C3B—N4B	1.3338 (17)
C3A—C31A	1.5206 (18)	C3B—C31B	1.5257 (18)
N4A—C5A	1.4619 (17)	N4B—C5B	1.4682 (16)
N4A—H4A	0.832 (16)	N4B—H4B	0.862 (16)
C5A—O51A	1.4089 (16)	C5B—O51B	1.3986 (16)
C5A—C54A	1.5533 (19)	C5B—C54B	1.5605 (18)
C21A—O21A	1.2009 (17)	C21B—O21B	1.2053 (18)
C21A—O22A	1.3442 (17)	C21B—O22B	1.3481 (17)
O22A—C23A	1.4558 (18)	O22B—C23B	1.4500 (19)
C23A—C24A	1.470 (3)	C23B—C24B	1.492 (3)
C23A—H23A	0.9900	C23B—H23C	0.9900
C23A—H23B	0.9900	C23B—H23D	0.9900
C24A—H24A	0.9800	C24B—H24D	0.9800
C24A—H24B	0.9800	C24B—H24E	0.9800
C24A—H24C	0.9800	C24B—H24F	0.9800
C31A—C32A	1.523 (2)	C31B—C33B	1.531 (2)
C31A—C34A	1.532 (2)	C31B—C34B	1.536 (2)
C31A—C33A	1.534 (2)	C31B—C32B	1.538 (2)
C32A—H32A	0.9800	C32B—H32D	0.9800
C32A—H32B	0.9800	C32B—H32E	0.9800
C32A—H32C	0.9800	C32B—H32F	0.9800
C33A—H33A	0.9800	C33B—H33D	0.9800
C33A—H33B	0.9800	C33B—H33E	0.9800
C33A—H33C	0.9800	C33B—H33F	0.9800
C34A—H34A	0.9800	C34B—H34D	0.9800
C34A—H34B	0.9800	C34B—H34E	0.9800
C34A—H34C	0.9800	C34B—H34F	0.9800
O51A—C52A	1.4398 (17)	O51B—C52B	1.4385 (17)
C52A—C53A	1.495 (2)	C52B—C53B	1.500 (2)
C52A—H52A	0.9900	C52B—H52C	0.9900
C52A—H52B	0.9900	C52B—H52D	0.9900
C53A—H53A	0.9800	C53B—H53D	0.9800
C53A—H53B	0.9800	C53B—H53E	0.9800
C53A—H53C	0.9800	C53B—H53F	0.9800
C54A—C56A	1.5317 (19)	C54B—C56B	1.5316 (19)
C54A—C57A	1.533 (2)	C54B—C55B	1.533 (2)
C54A—C55A	1.5374 (19)	C54B—C57B	1.5340 (19)
C55A—H55A	0.9800	C55B—H55D	0.9800
C55A—H55B	0.9800	C55B—H55E	0.9800
C55A—H55C	0.9800	C55B—H55F	0.9800
C56A—H56A	0.9800	C56B—H56D	0.9800
C56A—H56B	0.9800	C56B—H56E	0.9800
C56A—H56C	0.9800	C56B—H56F	0.9800
C57A—H57A	0.9800	C57B—H57D	0.9800
C57A—H57B	0.9800	C57B—H57E	0.9800
C57A—H57C	0.9800	C57B—H57F	0.9800
			
O1A—C1A—C2A	129.93 (12)	O1B—C1B—C2B	130.97 (13)
O1A—C1A—C5A	123.06 (12)	O1B—C1B—C5B	121.72 (12)
C2A—C1A—C5A	107.01 (11)	C2B—C1B—C5B	107.32 (11)
C3A—C2A—C1A	108.31 (11)	C3B—C2B—C1B	107.33 (11)
C3A—C2A—C21A	130.38 (12)	C3B—C2B—C21B	128.09 (12)
C1A—C2A—C21A	121.31 (12)	C1B—C2B—C21B	124.50 (12)
N4A—C3A—C2A	111.16 (12)	N4B—C3B—C2B	110.98 (12)
N4A—C3A—C31A	119.60 (11)	N4B—C3B—C31B	119.43 (12)
C2A—C3A—C31A	129.21 (12)	C2B—C3B—C31B	129.58 (12)
C3A—N4A—C5A	112.64 (11)	C3B—N4B—C5B	112.98 (11)
C3A—N4A—H4A	124.4 (10)	C3B—N4B—H4B	125.8 (11)
C5A—N4A—H4A	123.0 (11)	C5B—N4B—H4B	120.6 (11)
O51A—C5A—N4A	110.85 (11)	O51B—C5B—N4B	112.00 (10)
O51A—C5A—C54A	107.25 (10)	O51B—C5B—C1B	113.02 (11)
N4A—C5A—C54A	112.20 (11)	N4B—C5B—C1B	100.69 (10)
O51A—C5A—C1A	112.92 (11)	O51B—C5B—C54B	107.64 (10)
N4A—C5A—C1A	100.66 (10)	N4B—C5B—C54B	111.59 (11)
C54A—C5A—C1A	112.99 (11)	C1B—C5B—C54B	111.90 (11)
O21A—C21A—O22A	122.81 (13)	O21B—C21B—O22B	121.54 (13)
O21A—C21A—C2A	125.50 (13)	O21B—C21B—C2B	127.03 (13)
O22A—C21A—C2A	111.60 (12)	O22B—C21B—C2B	111.36 (12)
C21A—O22A—C23A	115.85 (12)	C21B—O22B—C23B	116.08 (13)
O22A—C23A—C24A	108.39 (15)	O22B—C23B—C24B	111.12 (17)
O22A—C23A—H23A	110.0	O22B—C23B—H23C	109.4
C24A—C23A—H23A	110.0	C24B—C23B—H23C	109.4
O22A—C23A—H23B	110.0	O22B—C23B—H23D	109.4
C24A—C23A—H23B	110.0	C24B—C23B—H23D	109.4
H23A—C23A—H23B	108.4	H23C—C23B—H23D	108.0
C23A—C24A—H24A	109.5	C23B—C24B—H24D	109.5
C23A—C24A—H24B	109.5	C23B—C24B—H24E	109.5
H24A—C24A—H24B	109.5	H24D—C24B—H24E	109.5
C23A—C24A—H24C	109.5	C23B—C24B—H24F	109.5
H24A—C24A—H24C	109.5	H24D—C24B—H24F	109.5
H24B—C24A—H24C	109.5	H24E—C24B—H24F	109.5
C3A—C31A—C32A	107.86 (12)	C3B—C31B—C33B	111.26 (12)
C3A—C31A—C34A	110.16 (12)	C3B—C31B—C34B	110.17 (11)
C32A—C31A—C34A	110.20 (14)	C33B—C31B—C34B	107.97 (13)
C3A—C31A—C33A	111.38 (12)	C3B—C31B—C32B	108.06 (11)
C32A—C31A—C33A	110.60 (15)	C33B—C31B—C32B	108.76 (13)
C34A—C31A—C33A	106.67 (13)	C34B—C31B—C32B	110.63 (12)
C31A—C32A—H32A	109.5	C31B—C32B—H32D	109.5
C31A—C32A—H32B	109.5	C31B—C32B—H32E	109.5
H32A—C32A—H32B	109.5	H32D—C32B—H32E	109.5
C31A—C32A—H32C	109.5	C31B—C32B—H32F	109.5
H32A—C32A—H32C	109.5	H32D—C32B—H32F	109.5
H32B—C32A—H32C	109.5	H32E—C32B—H32F	109.5
C31A—C33A—H33A	109.5	C31B—C33B—H33D	109.5
C31A—C33A—H33B	109.5	C31B—C33B—H33E	109.5
H33A—C33A—H33B	109.5	H33D—C33B—H33E	109.5
C31A—C33A—H33C	109.5	C31B—C33B—H33F	109.5
H33A—C33A—H33C	109.5	H33D—C33B—H33F	109.5
H33B—C33A—H33C	109.5	H33E—C33B—H33F	109.5
C31A—C34A—H34A	109.5	C31B—C34B—H34D	109.5
C31A—C34A—H34B	109.5	C31B—C34B—H34E	109.5
H34A—C34A—H34B	109.5	H34D—C34B—H34E	109.5
C31A—C34A—H34C	109.5	C31B—C34B—H34F	109.5
H34A—C34A—H34C	109.5	H34D—C34B—H34F	109.5
H34B—C34A—H34C	109.5	H34E—C34B—H34F	109.5
C5A—O51A—C52A	115.05 (10)	C5B—O51B—C52B	114.83 (10)
O51A—C52A—C53A	107.95 (12)	O51B—C52B—C53B	107.99 (13)
O51A—C52A—H52A	110.1	O51B—C52B—H52C	110.1
C53A—C52A—H52A	110.1	C53B—C52B—H52C	110.1
O51A—C52A—H52B	110.1	O51B—C52B—H52D	110.1
C53A—C52A—H52B	110.1	C53B—C52B—H52D	110.1
H52A—C52A—H52B	108.4	H52C—C52B—H52D	108.4
C52A—C53A—H53A	109.5	C52B—C53B—H53D	109.5
C52A—C53A—H53B	109.5	C52B—C53B—H53E	109.5
H53A—C53A—H53B	109.5	H53D—C53B—H53E	109.5
C52A—C53A—H53C	109.5	C52B—C53B—H53F	109.5
H53A—C53A—H53C	109.5	H53D—C53B—H53F	109.5
H53B—C53A—H53C	109.5	H53E—C53B—H53F	109.5
C56A—C54A—C57A	109.52 (12)	C56B—C54B—C55B	109.49 (12)
C56A—C54A—C55A	108.74 (12)	C56B—C54B—C57B	108.74 (12)
C57A—C54A—C55A	109.26 (12)	C55B—C54B—C57B	109.12 (12)
C56A—C54A—C5A	110.36 (12)	C56B—C54B—C5B	110.22 (11)
C57A—C54A—C5A	109.49 (11)	C55B—C54B—C5B	109.42 (11)
C55A—C54A—C5A	109.45 (11)	C57B—C54B—C5B	109.83 (11)
C54A—C55A—H55A	109.5	C54B—C55B—H55D	109.5
C54A—C55A—H55B	109.5	C54B—C55B—H55E	109.5
H55A—C55A—H55B	109.5	H55D—C55B—H55E	109.5
C54A—C55A—H55C	109.5	C54B—C55B—H55F	109.5
H55A—C55A—H55C	109.5	H55D—C55B—H55F	109.5
H55B—C55A—H55C	109.5	H55E—C55B—H55F	109.5
C54A—C56A—H56A	109.5	C54B—C56B—H56D	109.5
C54A—C56A—H56B	109.5	C54B—C56B—H56E	109.5
H56A—C56A—H56B	109.5	H56D—C56B—H56E	109.5
C54A—C56A—H56C	109.5	C54B—C56B—H56F	109.5
H56A—C56A—H56C	109.5	H56D—C56B—H56F	109.5
H56B—C56A—H56C	109.5	H56E—C56B—H56F	109.5
C54A—C57A—H57A	109.5	C54B—C57B—H57D	109.5
C54A—C57A—H57B	109.5	C54B—C57B—H57E	109.5
H57A—C57A—H57B	109.5	H57D—C57B—H57E	109.5
C54A—C57A—H57C	109.5	C54B—C57B—H57F	109.5
H57A—C57A—H57C	109.5	H57D—C57B—H57F	109.5
H57B—C57A—H57C	109.5	H57E—C57B—H57F	109.5
			
O1A—C1A—C2A—C3A	175.84 (14)	O1B—C1B—C2B—C3B	−171.89 (14)
C5A—C1A—C2A—C3A	−4.57 (15)	C5B—C1B—C2B—C3B	8.17 (14)
O1A—C1A—C2A—C21A	−4.3 (2)	O1B—C1B—C2B—C21B	11.2 (2)
C5A—C1A—C2A—C21A	175.27 (12)	C5B—C1B—C2B—C21B	−168.79 (12)
C1A—C2A—C3A—N4A	2.80 (16)	C1B—C2B—C3B—N4B	−5.04 (15)
C21A—C2A—C3A—N4A	−177.03 (13)	C21B—C2B—C3B—N4B	171.77 (13)
C1A—C2A—C3A—C31A	−175.43 (13)	C1B—C2B—C3B—C31B	173.79 (13)
C21A—C2A—C3A—C31A	4.7 (2)	C21B—C2B—C3B—C31B	−9.4 (2)
C2A—C3A—N4A—C5A	0.37 (16)	C2B—C3B—N4B—C5B	−0.50 (16)
C31A—C3A—N4A—C5A	178.79 (12)	C31B—C3B—N4B—C5B	−179.46 (11)
C3A—N4A—C5A—O51A	−122.73 (12)	C3B—N4B—C5B—O51B	125.64 (12)
C3A—N4A—C5A—C54A	117.39 (12)	C3B—N4B—C5B—C1B	5.27 (14)
C3A—N4A—C5A—C1A	−3.01 (14)	C3B—N4B—C5B—C54B	−113.61 (12)
O1A—C1A—C5A—O51A	−57.67 (17)	O1B—C1B—C5B—O51B	52.43 (17)
C2A—C1A—C5A—O51A	122.71 (12)	C2B—C1B—C5B—O51B	−127.62 (11)
O1A—C1A—C5A—N4A	−175.89 (13)	O1B—C1B—C5B—N4B	172.06 (12)
C2A—C1A—C5A—N4A	4.49 (13)	C2B—C1B—C5B—N4B	−7.99 (13)
O1A—C1A—C5A—C54A	64.28 (17)	O1B—C1B—C5B—C54B	−69.29 (16)
C2A—C1A—C5A—C54A	−115.35 (12)	C2B—C1B—C5B—C54B	110.66 (12)
C3A—C2A—C21A—O21A	−123.99 (18)	C3B—C2B—C21B—O21B	−20.3 (2)
C1A—C2A—C21A—O21A	56.2 (2)	C1B—C2B—C21B—O21B	155.96 (15)
C3A—C2A—C21A—O22A	59.31 (19)	C3B—C2B—C21B—O22B	162.64 (13)
C1A—C2A—C21A—O22A	−120.50 (14)	C1B—C2B—C21B—O22B	−21.06 (19)
O21A—C21A—O22A—C23A	4.9 (2)	O21B—C21B—O22B—C23B	0.2 (2)
C2A—C21A—O22A—C23A	−178.30 (14)	C2B—C21B—O22B—C23B	177.41 (14)
C21A—O22A—C23A—C24A	148.27 (16)	C21B—O22B—C23B—C24B	91.19 (19)
N4A—C3A—C31A—C32A	86.56 (17)	N4B—C3B—C31B—C33B	13.22 (18)
C2A—C3A—C31A—C32A	−95.34 (18)	C2B—C3B—C31B—C33B	−165.52 (14)
N4A—C3A—C31A—C34A	−33.74 (18)	N4B—C3B—C31B—C34B	132.93 (14)
C2A—C3A—C31A—C34A	144.35 (15)	C2B—C3B—C31B—C34B	−45.82 (19)
N4A—C3A—C31A—C33A	−151.90 (14)	N4B—C3B—C31B—C32B	−106.10 (14)
C2A—C3A—C31A—C33A	26.2 (2)	C2B—C3B—C31B—C32B	75.15 (18)
N4A—C5A—O51A—C52A	63.24 (15)	N4B—C5B—O51B—C52B	−52.86 (15)
C54A—C5A—O51A—C52A	−173.96 (11)	C1B—C5B—O51B—C52B	60.04 (15)
C1A—C5A—O51A—C52A	−48.84 (15)	C54B—C5B—O51B—C52B	−175.88 (11)
C5A—O51A—C52A—C53A	−161.21 (12)	C5B—O51B—C52B—C53B	−152.20 (12)
O51A—C5A—C54A—C56A	58.82 (14)	O51B—C5B—C54B—C56B	−173.80 (11)
N4A—C5A—C54A—C56A	−179.22 (11)	N4B—C5B—C54B—C56B	62.93 (14)
C1A—C5A—C54A—C56A	−66.26 (14)	C1B—C5B—C54B—C56B	−49.04 (15)
O51A—C5A—C54A—C57A	179.45 (11)	O51B—C5B—C54B—C55B	65.74 (14)
N4A—C5A—C54A—C57A	−58.60 (14)	N4B—C5B—C54B—C55B	−57.52 (15)
C1A—C5A—C54A—C57A	54.36 (15)	C1B—C5B—C54B—C55B	−169.49 (11)
O51A—C5A—C54A—C55A	−60.81 (14)	O51B—C5B—C54B—C57B	−54.01 (14)
N4A—C5A—C54A—C55A	61.15 (14)	N4B—C5B—C54B—C57B	−177.28 (11)
C1A—C5A—C54A—C55A	174.11 (11)	C1B—C5B—C54B—C57B	70.75 (14)

### Hydrogen-bond geometry (Å, º)

**Table:**

*D*—H···*A*	*D*—H	H···*A*	*D*···*A*	*D*—H···*A*
N4*A*—H4*A*···O1*B*^i^	0.832 (16)	2.175 (16)	3.0009 (15)	171.8 (14)
N4*B*—H4*B*···O1*A*	0.862 (16)	2.156 (17)	3.0089 (15)	170.4 (15)

Symmetry code: (i) *x*+1/2, −*y*+1/2, *z*+1/2.
